# Methyl 2-(5-chloro-1-methyl-2-oxo-2,3-di­hydro-1*H*-indol-3-ylidene)acetate

**DOI:** 10.1107/S1600536813011768

**Published:** 2013-05-11

**Authors:** Piskala Subburaman Kannan, PanneerSelvam Yuvaraj, Karthikeyan Manivannan, Boreddy S. R. Reddy, A. SubbiahPandi

**Affiliations:** a Department of Physics, S.M.K. Fomra Institute of Technology, Thaiyur, Chennai 603 103, India; b Industrial Chemistry Laboratory, Central Leather Research Institute, Adyar, Chennai 600 020, India; c Department of Physics, Presidency College (Autonomous), Chennai 600 005, India

## Abstract

The title compound, C_12_H_10_ClNO_3_, the indoline ring system is essentially planar, with a maximum deviation of 0.009 Å for the N atom. The indoline ring and acetate group are essentially coplanar, with a maximum deviation of 0.086 Å for the O atom. The mean plane through the methoxy­carbonyl­methyl group forms a dihedral angle of 3.68 (5)° with the plane of the indoline ring system. The mol­ecular structure is stabilized by an intra­molecular C—H⋯O hydrogen-bond inter­action. In the crystal, π–π stacking inter­actions [centroid–centroid distance = 3.7677 (8) Å] occur between benzene rings, forming a chain running along the *c*-axis direction.

## Related literature

For the biological activity of indole derivatives, see: Chai *et al.* (2006 ▶); Nieto *et al.* (2005 ▶); Singh *et al.* (2000 ▶); Andreani *et al.* (2001 ▶); Quetin-Leclercq (1994 ▶); Mukhopadhyay *et al.* (1981 ▶); Taylor *et al.* (1999 ▶). For closely related structures, see: Hu *et al.* (2011 ▶); Han & Luo (2012 ▶); Deng (2011 ▶). For puckering parameters, see: Cremer & Pople (1975 ▶).
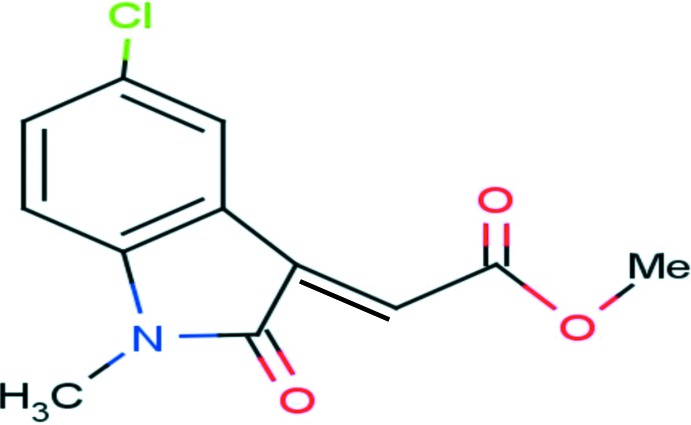


## Experimental

### 

#### Crystal data

C_12_H_10_ClNO_3_
*M* *_r_* = 251.66
Monoclinic, 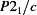

*a* = 8.4709 (7) Å
*b* = 17.1658 (13) Å
*c* = 7.9481 (6) Å
β = 107.228 (4)°
*V* = 1103.88 (15) Å^3^
*Z* = 4
Mo *K*α radiation
μ = 0.34 mm^−1^
*T* = 293 K
0.30 × 0.25 × 0.20 mm

#### Data collection

Bruker SMART APEXII area-detector diffractometer
Absorption correction: multi-scan (*SADABS*; Bruker, 2008 ▶) *T*_min_ = 0.903, *T*_max_ = 0.934
10447 measured reflections
2815 independent reflections
2410 reflections with *I* > 2σ(*I*)
*R*_int_ = 0.031

#### Refinement

*R*[*F* ^2^ > 2σ(*F* ^2^)] = 0.038
*wR*(*F* ^2^) = 0.138
*S* = 1.13
2815 reflections
157 parameters
H-atom parameters constrained
Δρ_max_ = 0.34 e Å^−3^
Δρ_min_ = −0.21 e Å^−3^

### 

Data collection: *APEX2* (Bruker, 2008 ▶); cell refinement: *SAINT* (Bruker, 2008 ▶); data reduction: *SAINT*; program(s) used to solve structure: *SHELXS97* (Sheldrick, 2008 ▶); program(s) used to refine structure: *SHELXL97* (Sheldrick, 2008 ▶); molecular graphics: *ORTEP-3 for Windows* (Farrugia, 2012 ▶); software used to prepare material for publication: *SHELXL97* and *PLATON* (Spek, 2009 ▶).

## Supplementary Material

Crystal structure: contains datablock(s) global, I. DOI: 10.1107/S1600536813011768/bx2438sup1.cif

Structure factors: contains datablock(s) I. DOI: 10.1107/S1600536813011768/bx2438Isup2.hkl

Supplementary material file. DOI: 10.1107/S1600536813011768/bx2438Isup3.cml

Additional supplementary materials:  crystallographic information; 3D view; checkCIF report

## Figures and Tables

**Table 1:** Hydrogen-bond geometry (Å, °)

*D*—H⋯*A*	*D*—H	H⋯*A*	*D*⋯*A*	*D*—H⋯*A*
C6—H6⋯O2	0.93	2.30	3.0181 (17)	134

**Fig. 1.**
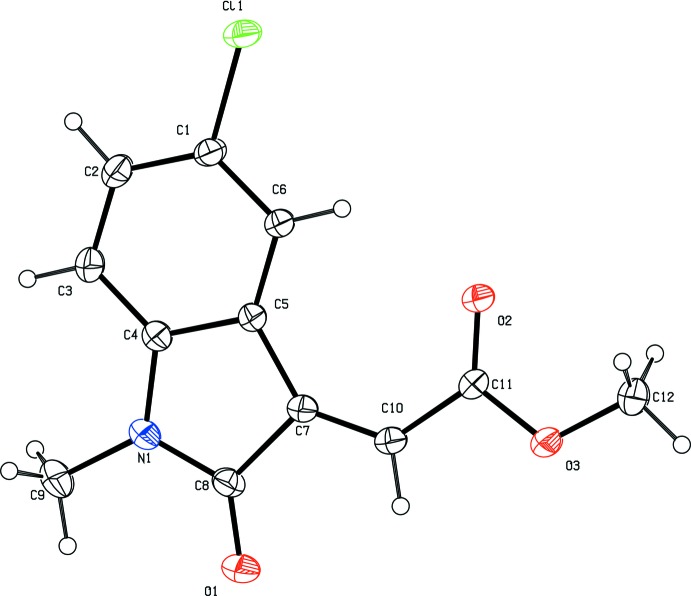
The structure of showing the atom-numbering scheme. The displacement ellipsoids are drawn at the 30% probability level.

## supplementary crystallographic information

### Comment

Indole derivatives exhibit antihepatitis B virus (Chai *et al.*, 2006) and antibacterial (Nieto *et al.*, 2005) activities. Indole derivatives have been found to exhibit antibacterial, antifungal (Singh *et al.*, 2000) and antitumour activities (Andreani *et al.*, 2001). Some of the indole alkaloids extracted from plants possess interesting cytotoxic, antitumour or antiparasitic properties (Quetin-Leclercq, 1994; Mukhopadhyay *et al.*, 1981). Pyrido[1,2-a]indole derivatives have been identified as potent inhibitors of human immunodeficiency virus type 1 (Taylor *et al.*, 1999).

In the title compound, C_12_H_10_Cl_1_N_1_O_3_,Figure 1 the indole ring system [C1-C8/N1] is essentially co-planar, with maxmium deviations of -0.009 Å for N1 atom. The indole ring and acetate group systems are essentially co-planar, with maxmium deviations of 0.086 Å for O2 atom. The mean plane through the methyl 4-oxobutanoate(acetate) group forms a dihedral angle of 3.68 (5)° with the plane of the indole ring system.

The geometric parameters of the title molecule (Fig. 1) agree well with the reported similar structures (Han *et al.*, 2012). The sum of the angles at N1 [359.98 (1)°] of the pyrrolidine rings are in accordance with *sp*^2^ hybridizations.The crystal structure is stabilized by intramolecular C-H···O hydrogen bond interaction and π -π stacking interactions [centroid–centroid distance = 3.7677 (8) Å] between benzene rings [Cg1–Cg1^i^ (Cg1:C1–C6) (i):symmetry code: -x,1-y,1-z]

### Experimental

To a mixture of 1eq of (*E*)-methyl 2-(5-chloro-1-methyl-2-oxoindolin- 3-ylidene) acetate, 1eq of isatin and 1.5eq of sarcosine were dissolved in acetonitrile. This reaction mixture refluxed at 80°C for 8hours. Completion of reaction monitor by Thin layar chromatography. The reaction mixture was extracted with ethyl acetate and water. The product was dried and purified by coloumn chromatography using ethyl acetate and hexane (1:9) as an elutent to afford pure Dispiro oxindole. Yield (90%). Single crystals suitable for X-ray diffraction were obtained by slow evaporation of a solution of the title compound in ethyl acetate at room temperature.

### Refinement

All H atoms were fixed geometrically and allowed to ride on their parent C atoms, with C—H distances fixed in the range 0.93–0.97 Å with *U*_iso_(H) = 1.5*U*_eq_(C) for methyl H 1.2*U*_eq_(C) for other H atoms. The positions of methyl hydrogens were optimized rotationally.

### Crystal data

**Table:**

C_12_H_10_ClNO_3_	*F*(000) = 520
*M**_r_* = 251.66	*D*_x_ = 1.514 Mg m^−^^3^
Monoclinic, *P*2_1_/*c*	Mo *K*α radiation, λ = 0.71073 Å
Hall symbol: -P 2ybc	Cell parameters from 2857 reflections
*a* = 8.4709 (7) Å	θ = 2.4–28.6°
*b* = 17.1658 (13) Å	µ = 0.34 mm^−^^1^
*c* = 7.9481 (6) Å	*T* = 293 K
β = 107.228 (4)°	Block, colourless
*V* = 1103.88 (15) Å^3^	0.30 × 0.25 × 0.20 mm
*Z* = 4	

### Data collection

**Table:**

Bruker SMART APEXII area-detector diffractometer	2815 independent reflections
Radiation source: fine-focus sealed tube	2410 reflections with *I* > 2σ(*I*)
Graphite monochromator	*R*_int_ = 0.031
ω and φ scans	θ_max_ = 28.6°, θ_min_ = 2.4°
Absorption correction: multi-scan (*SADABS*; Bruker, 2008)	*h* = −11→11
*T*_min_ = 0.903, *T*_max_ = 0.934	*k* = −23→23
10447 measured reflections	*l* = −10→10

### Refinement

**Table:**

Refinement on *F*^2^	Secondary atom site location: difference Fourier map
Least-squares matrix: full	Hydrogen site location: inferred from neighbouring sites
*R*[*F*^2^ > 2σ(*F*^2^)] = 0.038	H-atom parameters constrained
*wR*(*F*^2^) = 0.138	*w* = 1/[σ^2^(*F*_o_^2^) + (0.1*P*)^2^] where *P* = (*F*_o_^2^ + 2*F*_c_^2^)/3
*S* = 1.13	(Δ/σ)_max_ < 0.001
2815 reflections	Δρ_max_ = 0.34 e Å^−^^3^
157 parameters	Δρ_min_ = −0.21 e Å^−^^3^
0 restraints	Extinction correction: *SHELXL97* (Sheldrick, 2008), Fc^*^=kFc[1+0.001xFc^2^λ^3^/sin(2θ)]^-1/4^
Primary atom site location: structure-invariant direct methods	Extinction coefficient: 0.008 (3)

### Special details

**Table:**

Geometry. All esds (except the esd in the dihedral angle between two l.s. planes) are estimated using the full covariance matrix. The cell esds are taken into account individually in the estimation of esds in distances, angles and torsion angles; correlations between esds in cell parameters are only used when they are defined by crystal symmetry. An approximate (isotropic) treatment of cell esds is used for estimating esds involving l.s. planes.
Refinement. Refinement of F^2^ against ALL reflections. The weighted R-factor wR and goodness of fit S are based on F^2^, conventional R-factors R are based on F, with F set to zero for negative F^2^. The threshold expression of F^2^ > 2sigma(F^2^) is used only for calculating R-factors(gt) etc. and is not relevant to the choice of reflections for refinement. R-factors based on F^2^ are statistically about twice as large as those based on F, and R- factors based on ALL data will be even larger.

### Fractional atomic coordinates and isotropic or equivalent isotropic displacement parameters (Å^2^)

**Table:**

	*x*	*y*	*z*	*U*_iso_*/*U*_eq_	
C1	0.18640 (15)	0.52152 (8)	0.41988 (16)	0.0337 (3)	
C2	0.22645 (18)	0.44379 (9)	0.44263 (18)	0.0400 (3)	
H2	0.3224	0.4283	0.5281	0.048*	
C3	0.12324 (18)	0.38846 (8)	0.33765 (19)	0.0396 (3)	
H3	0.1487	0.3357	0.3514	0.047*	
C4	−0.01720 (16)	0.41357 (7)	0.21330 (17)	0.0321 (3)	
C5	−0.05973 (15)	0.49301 (7)	0.18943 (15)	0.0287 (3)	
C6	0.04383 (15)	0.54819 (7)	0.29523 (15)	0.0317 (3)	
H6	0.0187	0.6010	0.2831	0.038*	
C7	−0.21570 (16)	0.49743 (7)	0.04971 (15)	0.0306 (3)	
C8	−0.25984 (18)	0.41402 (7)	−0.00755 (18)	0.0344 (3)	
C9	−0.1339 (2)	0.28462 (9)	0.0829 (2)	0.0509 (4)	
H9A	−0.2332	0.2668	−0.0022	0.076*	
H9B	−0.1267	0.2625	0.1960	0.076*	
H9C	−0.0398	0.2687	0.0476	0.076*	
C10	−0.31915 (15)	0.55312 (8)	−0.03603 (16)	0.0349 (3)	
H10	−0.4126	0.5363	−0.1232	0.042*	
C11	−0.30207 (15)	0.63753 (7)	−0.00835 (16)	0.0341 (3)	
C12	−0.4227 (2)	0.75770 (8)	−0.1167 (2)	0.0499 (4)	
H12A	−0.4311	0.7724	−0.0031	0.075*	
H12B	−0.5155	0.7782	−0.2068	0.075*	
H12C	−0.3222	0.7783	−0.1314	0.075*	
N1	−0.13690 (14)	0.36835 (6)	0.09414 (14)	0.0370 (3)	
O1	−0.38178 (14)	0.39168 (7)	−0.12194 (15)	0.0484 (3)	
O2	−0.19597 (13)	0.67151 (6)	0.10265 (16)	0.0503 (3)	
O3	−0.42172 (12)	0.67452 (6)	−0.12966 (13)	0.0426 (3)	
Cl1	0.31706 (4)	0.58922 (2)	0.55565 (4)	0.04557 (17)	

### Atomic displacement parameters (Å^2^)

**Table:**

	*U*^11^	*U*^22^	*U*^33^	*U*^12^	*U*^13^	*U*^23^
C1	0.0283 (6)	0.0363 (7)	0.0319 (6)	−0.0017 (5)	0.0015 (5)	0.0008 (4)
C2	0.0347 (7)	0.0386 (7)	0.0406 (6)	0.0058 (6)	0.0016 (5)	0.0059 (5)
C3	0.0409 (7)	0.0302 (6)	0.0437 (7)	0.0054 (5)	0.0065 (6)	0.0047 (5)
C4	0.0331 (7)	0.0284 (6)	0.0340 (6)	−0.0013 (4)	0.0084 (5)	−0.0007 (4)
C5	0.0277 (6)	0.0265 (6)	0.0306 (5)	0.0001 (4)	0.0065 (4)	−0.0003 (4)
C6	0.0285 (6)	0.0303 (6)	0.0324 (6)	−0.0003 (5)	0.0031 (4)	−0.0001 (4)
C7	0.0289 (6)	0.0299 (6)	0.0307 (6)	−0.0030 (4)	0.0052 (4)	−0.0027 (4)
C8	0.0372 (7)	0.0298 (6)	0.0346 (6)	−0.0055 (5)	0.0084 (5)	−0.0045 (4)
C9	0.0618 (10)	0.0273 (7)	0.0595 (9)	−0.0019 (6)	0.0116 (7)	−0.0034 (6)
C10	0.0298 (6)	0.0344 (7)	0.0346 (6)	−0.0026 (5)	0.0003 (5)	−0.0025 (5)
C11	0.0298 (6)	0.0345 (7)	0.0332 (6)	0.0040 (5)	0.0021 (4)	0.0012 (5)
C12	0.0546 (9)	0.0351 (8)	0.0501 (8)	0.0081 (6)	0.0003 (7)	0.0075 (6)
N1	0.0406 (6)	0.0272 (6)	0.0399 (6)	−0.0034 (4)	0.0068 (5)	−0.0045 (4)
O1	0.0443 (6)	0.0404 (5)	0.0503 (6)	−0.0094 (4)	−0.0018 (5)	−0.0099 (4)
O2	0.0423 (6)	0.0347 (5)	0.0547 (6)	0.0022 (4)	−0.0151 (4)	−0.0065 (4)
O3	0.0408 (6)	0.0336 (5)	0.0406 (5)	0.0029 (4)	−0.0074 (4)	0.0021 (4)
Cl1	0.0370 (2)	0.0450 (3)	0.0434 (2)	−0.00716 (13)	−0.00546 (16)	−0.00314 (13)

### Geometric parameters (Å, º)

**Table:**

C1—C2	1.3753 (19)	C8—O1	1.2183 (18)
C1—C6	1.3932 (16)	C8—N1	1.3615 (18)
C1—Cl1	1.7415 (13)	C9—N1	1.4408 (18)
C2—C3	1.389 (2)	C9—H9A	0.9600
C2—H2	0.9300	C9—H9B	0.9600
C3—C4	1.3717 (18)	C9—H9C	0.9600
C3—H3	0.9300	C10—C11	1.4661 (19)
C4—N1	1.3995 (16)	C10—H10	0.9300
C4—C5	1.4089 (17)	C11—O2	1.2070 (16)
C5—C6	1.3917 (16)	C11—O3	1.3346 (15)
C5—C7	1.4545 (17)	C12—O3	1.4320 (16)
C6—H6	0.9300	C12—H12A	0.9600
C7—C10	1.3389 (19)	C12—H12B	0.9600
C7—C8	1.5153 (17)	C12—H12C	0.9600
			
C2—C1—C6	122.71 (12)	N1—C8—C7	106.77 (11)
C2—C1—Cl1	118.64 (10)	N1—C9—H9A	109.5
C6—C1—Cl1	118.64 (10)	N1—C9—H9B	109.5
C1—C2—C3	119.83 (13)	H9A—C9—H9B	109.5
C1—C2—H2	120.1	N1—C9—H9C	109.5
C3—C2—H2	120.1	H9A—C9—H9C	109.5
C4—C3—C2	118.36 (12)	H9B—C9—H9C	109.5
C4—C3—H3	120.8	C7—C10—C11	127.50 (11)
C2—C3—H3	120.8	C7—C10—H10	116.3
C3—C4—N1	127.82 (11)	C11—C10—H10	116.3
C3—C4—C5	122.27 (12)	O2—C11—O3	122.67 (12)
N1—C4—C5	109.92 (11)	O2—C11—C10	127.33 (11)
C6—C5—C4	119.15 (11)	O3—C11—C10	109.98 (11)
C6—C5—C7	133.90 (11)	O3—C12—H12A	109.5
C4—C5—C7	106.94 (11)	O3—C12—H12B	109.5
C5—C6—C1	117.68 (11)	H12A—C12—H12B	109.5
C5—C6—H6	121.2	O3—C12—H12C	109.5
C1—C6—H6	121.2	H12A—C12—H12C	109.5
C10—C7—C5	137.34 (12)	H12B—C12—H12C	109.5
C10—C7—C8	117.12 (12)	C8—N1—C4	110.84 (10)
C5—C7—C8	105.52 (11)	C8—N1—C9	124.20 (12)
O1—C8—N1	126.31 (12)	C4—N1—C9	124.94 (12)
O1—C8—C7	126.92 (13)	C11—O3—C12	116.21 (11)
			
C6—C1—C2—C3	0.6 (2)	C5—C7—C8—O1	179.90 (14)
Cl1—C1—C2—C3	179.16 (10)	C10—C7—C8—N1	178.76 (11)
C1—C2—C3—C4	−0.1 (2)	C5—C7—C8—N1	0.12 (13)
C2—C3—C4—N1	179.68 (12)	C5—C7—C10—C11	−0.4 (2)
C2—C3—C4—C5	−0.2 (2)	C8—C7—C10—C11	−178.48 (12)
C3—C4—C5—C6	0.01 (19)	C7—C10—C11—O2	−4.1 (2)
N1—C4—C5—C6	−179.87 (10)	C7—C10—C11—O3	174.29 (13)
C3—C4—C5—C7	−179.19 (12)	O1—C8—N1—C4	−179.33 (13)
N1—C4—C5—C7	0.93 (13)	C7—C8—N1—C4	0.45 (14)
C4—C5—C6—C1	0.42 (17)	O1—C8—N1—C9	−0.9 (2)
C7—C5—C6—C1	179.36 (11)	C7—C8—N1—C9	178.84 (12)
C2—C1—C6—C5	−0.71 (19)	C3—C4—N1—C8	179.24 (13)
Cl1—C1—C6—C5	−179.32 (9)	C5—C4—N1—C8	−0.88 (14)
C6—C5—C7—C10	2.1 (2)	C3—C4—N1—C9	0.9 (2)
C4—C5—C7—C10	−178.84 (15)	C5—C4—N1—C9	−179.26 (13)
C6—C5—C7—C8	−179.66 (13)	O2—C11—O3—C12	−2.8 (2)
C4—C5—C7—C8	−0.63 (12)	C10—C11—O3—C12	178.73 (12)
C10—C7—C8—O1	−1.5 (2)		

### Hydrogen-bond geometry (Å, º)

**Table:**

*D*—H···*A*	*D*—H	H···*A*	*D*···*A*	*D*—H···*A*
C6—H6···O2	0.93	2.30	3.0181 (17)	134
